# T1 vs. T2 weighted magnetic resonance imaging to assess total kidney volume in patients with autosomal dominant polycystic kidney disease

**DOI:** 10.1007/s00261-017-1285-2

**Published:** 2017-09-04

**Authors:** Maatje D. A. van Gastel, A. Lianne Messchendorp, Peter Kappert, Merel A. Kaatee, Marissa de Jong, Remco J. Renken, Gert J. ter Horst, Shekar V. K. Mahesh, Ron T. Gansevoort, On behalf of the DIPAK Consortium

**Affiliations:** 1 Department of Nephrology, University Medical Center Groningen, University of Groningen, PO Box 30.001, 9700 RB Groningen, The Netherlands; 2 Department of Radiology, University Medical Center Groningen, University of Groningen, Groningen, The Netherlands; 3 Center for Medical Imaging, University Medical Center Groningen, University of Groningen, Groningen, The Netherlands; 4 Neuro Imaging Center, University Medical Center Groningen, University of Groningen, Groningen, The Netherlands

**Keywords:** T1, T2, MRI, ADPKD, Polycystic kidney disease, Total kidney volume

## Abstract

**Purpose:**

In ADPKD patients total kidney volume (TKV) measurement using MRI is performed to predict rate of disease progression. Historically T1 weighted images (T1) were used, but the methodology of T2 weighted imaging (T2) has evolved. We compared the performance of both sequences.

**Methods:**

40 ADPKD patients underwent an abdominal MRI at baseline and follow-up. TKV was measured by manual tracing with Analyze Direct 11.0 software. Three readers established intra- and interreader coefficients of variation (CV). T1 and T2 measured kidney volumes and growth rates were compared with ICC and Bland–Altman analyses.

**Results:**

Participants were 49.7 ± 7.0 years of age, 55.0% female, with estimated GFR of 50.1 ± 11.5 mL/min/1.73 m^2^. CVs were low and comparable for T2 and T1 (intrareader: 0.83% [0.48–1.79] vs. 1.15% [0.34–1.77], *P* = 0.9, interreader: 2.18% [1.59–2.61] vs. 1.69% [1.07–3.87], *P* = 0.9). TKV was clinically similar, but statistically significantly different between T2 and T1: 1867 [1172–2721] vs. 1932 [1180–2551] mL, respectively (*P* = 0.006), with a bias of only 0.8% and high agreement (ICC 0.997). Percentage kidney growth during 2.2 ± 0.3 years was similar for T2 and T1 (9.3 ± 10.6% vs. 7.8 ± 9.9%, *P* = 0.1, respectively), with a bias of 1.5% and high agreement (ICC 0.843). T2 was more often of sufficient quality for volume measurement (86.7% vs. 71.1%, *P* < 0.001).

**Conclusions:**

In patients with ADPKD, measurement of kidney volume and growth rate performs similarly when using T2 compared to T1 weighted images, although T2 performs better on secondary outcome parameters; they are more often of sufficient quality for volume measurement and result in slightly lower intra- and interreader variability.

**Electronic supplementary material:**

The online version of this article (doi:10.1007/s00261-017-1285-2) contains supplementary material, which is available to authorized users.

Autosomal Dominant Polycystic Kidney Disease (ADPKD) is the most common hereditary renal disease, with a prevalence of 3–4 per 10,000 in the general population [1, 2]. The disease is characterized by cyst formation in both kidneys, leading to pain, hematuria, and renal function loss. Seventy percent of the affected patients reach end-stage renal disease between the fourth and seventh decade of life [3].

Monitoring glomerular filtration rate to assess disease severity and progression in ADPKD has limitations, because GFR can remain within the normal range for prolonged periods of time, despite nephron loss, due to hyperfiltration of residual nephrons [4]. Measurement of total kidney volume (TKV) has been shown to be a reliable surrogate for assessment of disease severity and progression in these patients, with MR imaging being superior to ultrasonography, particularly for the visualization of small renal cysts [5, 6].

Historically, gadolinium enhanced T1 weighted images were used for the measurement of TKV because of the short scanning time, low variations in image quality and high contrast of the renal structures against the surrounding tissues [7]. In patients with impaired kidney function the use of gadolinium is avoided, because exposure to this contrast agent has been found to be associated with a higher incidence of nephrogenic systemic fibrosis [8], although the risk for this complication is probably lower than previously assumed [9]. When not using gadolinium contrast, T2 weighted images might be preferred over T1 weighted images for the measurement of the TKV, because this technique shows high kidney tissue contrast and hyperintense renal cysts, that may help to better delineate the kidney boundaries against background tissue [5]. In the past, T2 weighted imaging required longer scanning time and multiple breath-hold scanning, and was more prone to misregistration, motion artifacts and heterogeneous tissue signal intensities leading to high variation in scanning quality [7]. For that reason non-gadolinium-enhanced T1-MR imaging has become the preferred method to assess TKV. However, the single-shot T2 weighted techniques have evolved over the last decades, making T2 weighted imaging potentially preferable over T1 weighted imaging for TKV measurement. For instance, the single-shot fast spin-echo T2 technique has a shorter examination time, fewer motion artifacts, and the technique ensures that all images are obtained from the same anatomic position regardless of the patients’ ability to hold their breath [10].

We compared the performance of using T2 and T1 weighted MR images for the measurement of kidney volume and growth in patients with ADPKD, and tested the hypothesis that the use of T2 might be preferred over T1 weighted images.

## Materials and methods

### Patients and study design

For this study MR images were used of a subset of ADPKD patients that participated in the DIPAK-1 study, a randomized controlled trial in which the efficacy of lanreotide to halt disease progression in ADPKD is assessed. Patients diagnosed with ADPKD based on the revised Ravine criteria [11], aged 18–60 years, with an estimated glomerular filtration rate (eGFR) of 30–60 mL/min/1.73 m^2^ were included between 2012 and 2015 at the University Medical Centers of Groningen (UMCG), Leiden (LUMC), Nijmegen (Radboud UMC) and Rotterdam (Erasmus MC), all in the Netherlands. Details of the study protocol have been published elsewhere [12]. The Medical Ethical Committee of the UMC Groningen approved the protocol of this study that was conducted in accordance with the International Conference of Harmonization Good Clinical Practice Guidelines and in adherence to the ethical principles that have their origin in the Declaration of Helsinki. Informed consent was obtained from all individual participants included in the study.

### Magnetic resonance imaging

All participants underwent a standardized abdominal MRI without the use of intravenous contrast. All MR images were made using a 1.5 or 3.0 Tesla MRI scanner. Five different MRI scanners were used: (1) Magnetom Avanto, Siemens, Erlangen, Germany; (2) Ingenia, Philips, Eindhoven, the Netherlands; (3) GE Medical Systems, Buckinghamshire, United Kingdom; (4) Intera, Philips, Eindhoven, the Netherlands; and (5) Magnetom TRIO, Siemens, Erlangen, Germany. Coils were placed onto the anterior and posterior abdominal walls directly over the kidneys. A short scout was scanned to localize the kidneys. Subsequently two series of images were scanned for these analyses. First a coronal T2 single-shot fast spin echo was scanned (slice thickness 4 mm, gap/spacing 0 mm, FOV 35 cm, matrix 256 × 256, flip angle = 40°–50°, and different TR’s and TE’s (always in-phase) per brand MRI scanner: TE ≈ 100 ms for Siemens, TE ≈ 190 ms and TR ≈ max. 1400 ms for GE and TE ≈ 70 ms and TR ≈ max. 1900 ms for Philips). Thereafter a coronal T1-3D spoiled gradient echo was made (same characteristics except TE ≈ 2 ms, TR ≈ 4 ms and flip angle ≤15°). When a 35 cm FOV was insufficient, the FOV could be increased. The first and the last slice of the scan had to be an image without kidney tissue, to ensure whole organ imaging. The obtained MR images were anonymized and sent via a secured server to the central reading facility at the UMC Groningen, where kidney volumes were measured. The three readers were specifically trained to measure TKV on both T1 as well as T2 weighted images. During their training period, they measured 15 MR images per sequence, thus 30 kidney volumes, under supervision and guidance of an experienced MRI technician using a standard operating procedure. After these readers completed their training, they were allowed to measure TKV. This protocol was implemented in 2012 and was not changed during the study to avoid systematic bias.

### Measuring kidney volume on T1 and T2 weighted images

**Fig. 1:**
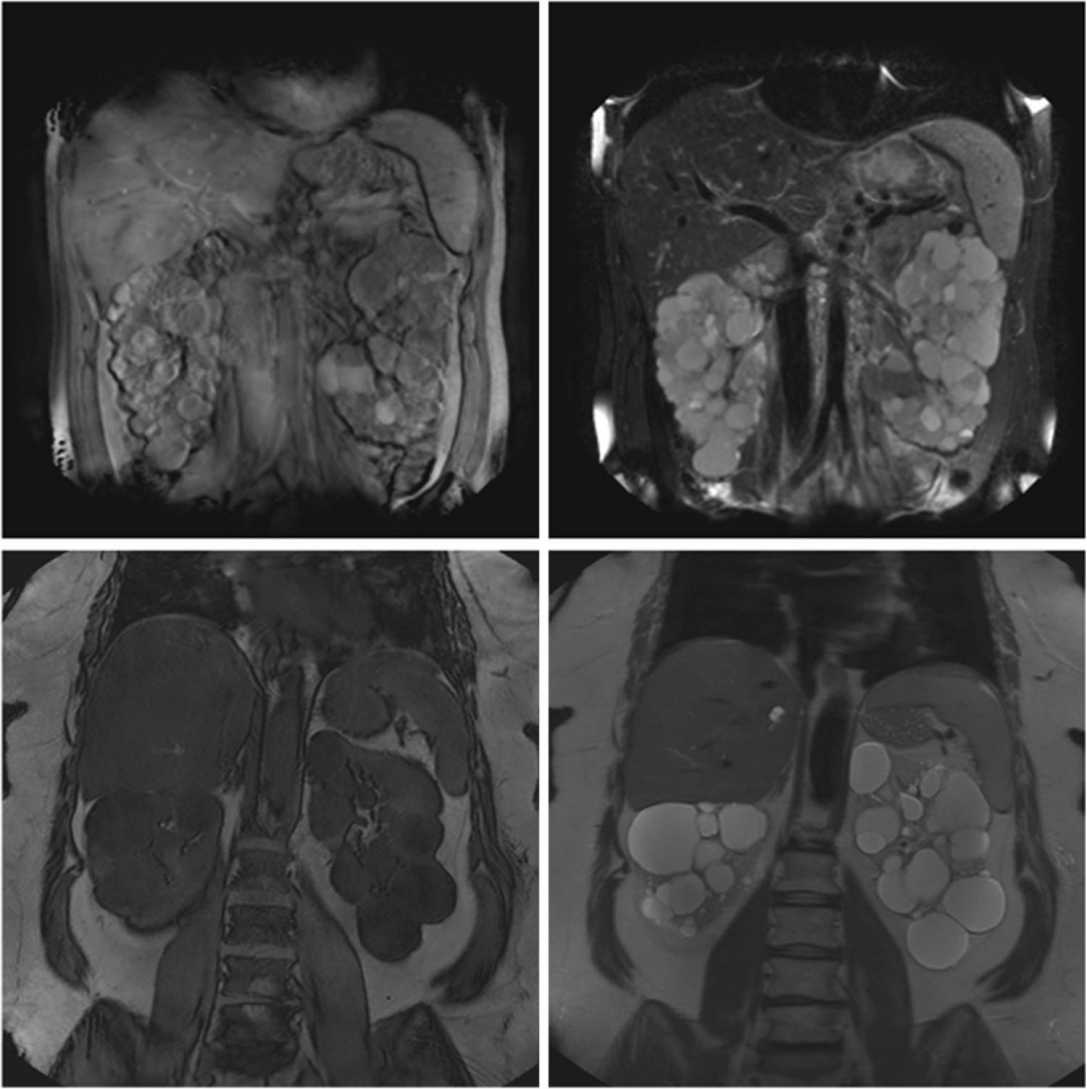
Examples of the T1-3D spoiled gradient echo (*left*) and T2 single-shot fast spin echo (*right*), examples from two different patients on two different scanners

Kidney volumes were measured on the coronal T2 single-shot fast spin-echo sequence and the coronal T1-3D spoiled gradient echo (Fig. 1). If one of those sequences showed too low quality, the patient was not eligible for these analyses. The assessment of quality was based upon the judgment of one reader whether or not the kidney boundaries were manually traceable. This was predominantly based on the appearance of motion artifacts. When the quality of the image was deemed too low, another reader was asked to confirm this assessment. The kidney boundaries were manually traced using the commercially available software Analyze Direct 11.0 (Analyze Direct, Inc., Overland Park, KS, USA). The kidney volumes were calculated from the set of contiguous images by summing the products of the area measurements within the kidney boundaries and slice thickness. Non-renal parenchyma, e.g., the renal hilus, was excluded from measurement. Importantly, all measurements were performed by readers blinded for patient number and previous TKV measurements.

### Statistical analysis

Baseline characteristics of the study population are given. Data are provided as mean with standard deviation (SD), or as median with interquartile range [IQR] in case of non-normal distribution.

In a test set of 12 patients kidney volumes were measured twice by three readers. This test set was used to assess the intra- and interreader reliability. Three MR images per MRI scanner were selected. Kidney volumes ranged from approximately 670 to 4000 mL. The intra- and interreader reliability for the left, right, and total kidney volume were assessed using the intraclass correlation coefficient (ICC). Reproducibility was evaluated by assessing intra- and interreader coefficient of variability (CV). Intrareader CV was calculated per MR image for each of the readers as the standard deviation of the two TKV values divided by the mean TKV multiplied by 100%. Interreader CV was calculated for each of the 12 MR images as the standard deviation of TKV values assessed by all three readers divided by the mean TKV of that image multiplied by 100%. As subgroup analysis intra- and interreader CVs were calculated for the different MRI scanners separately and for quartiles of T1 breath-hold trigger time.

To investigate whether the TKV results obtained with T1 and T2 weighted images correlate, orthogonal regression analysis was performed, and the ICC was calculated using all MR images of our cohort. Agreement between T1 and T2 measurements was evaluated by Bland–Altman analyses, where bias and precision are defined as mean difference and SD of the mean difference. Subsequently, serial MR images of 40 patients were used to determine whether both methods can accurately detect changes in TKV. Correlation between changes in TKV measured using T1 and T2 weighted images was assessed similarly using orthogonal regression analysis, calculation of ICC, and Bland–Altman analyses. Follow-up scans were preferably performed on the same MRI scanner as at baseline, and TKV was measured using the same series of images as at baseline. As sensitivity analyses we tested whether differences in kidney volumes (cross-sectionally) and growth rate (longitudinally) between T1 and T2 were dependent on the type of MRI scanner, T1 breath-hold trigger time, or on height-adjusted total liver volume, according to the polycystic liver disease (PLD) classification [13].

To assess the consequences of using T2 instead of T1 weighted images, four analyses were performed. First, the effect on classification according to Mayo height-adjusted TKV (htTKV) risk class [14], and second, the consequences for the sample size calculation of clinical trials were assessed (assuming a power of 80% and a 2-sided *α* of 0.05). Third, we analyzed the percentage of MR images that was deemed sufficient for TKV measurement. Fourth, we compared the duration of TKV measurement using T2 and T1 weighted images.

Differences in paired non-parametric data were tested with a Wilcoxon signed-rank test and in paired parametric data with a paired *T* test. A one sample *T* test was used when analyzing percentage difference of volumes measured on T2 compared to T1 weighted images, taking the volumes on T1 weighted images as 100%. For testing between more than two groups, a Kruskal–Wallis test was used for non-parametric data and an ANOVA for parametric data with Bonferroni correction. A *χ* ^2^ test was used for differences between proportions. All analyses were performed with SPSS, version 22.0 (SPSS Inc). *P* < 0.05 was considered to be statistically significant.

## Results

### Patient characteristics

Of the 40 included patients, 55.0% was female and the average age was 49.7 ± 7.0 years. Estimated GFR was 50.1 ± 11.5 mL/min/1.73 m^2^. The mean systolic and diastolic blood pressure values were 135 ± 13 and 83 ± 9 mmHg, respectively. Of all patients 97.5% used antihypertensive medication of which 87.5% used a RAAS blocker. Patients had a follow-up of 2.2 ± 0.3 years.

### Intra- and interreader reliability

The intra- and interreader reliability for both the T1 and T2 weighted images were high, with ICCs ranging from 0.997 to 1.000. Although the intra- and interreader CVs of both T1 and T2 weighted images were comparable for TKV, the intra- and interreader CVs for the separate kidneys were significantly lower for T2 (Table 1). There were no significant differences in intra- and interreader CVs between the right and left kidney, neither for T1 (*P* = 0.1 and *P* = 0.5, respectively) nor for T2 (*P* = 0.5 and *P* = 0.8, respectively) weighted images. No significant differences in intra- or interreader CVs were observed between the different MRI scanners or according to T1 breath-hold trigger time for the left, right, or total kidney volume (Supplementary Tables 1 and 6).

**Table 1:** Intra- and interreader coefficients of variability in kidney volume measurements when using T1 or T2 weighted images (three readers)

	Intrareader CV (%)		Interreader CV (%)	
	T1	T2	T1	T2
Left kidney	0.94 [0.52–1.65]	0.62 [0.25–0.87]*	1.62 [1.12–3.57]	1.00 [0.89–1.52]*
Right kidney	1.46 [0.71–2.00]	0.63 [0.25–1.04]*	2.63 [0.88–4.10]	1.25 [0.80–1.88]*
Total kidney	1.15 [0.34–1.77]	0.83 [0.48–1.79]	1.69 [1.07–3.87]	2.18 [1.59–2.61]

Values are given as median [IQR]. *P* values were calculated using a paired Wilcoxon signed-rank test

*CV* coefficient of variability

*P < 0.05

### Comparing T1 and T2 weighted images to measure kidney volume

**Fig. 2:**
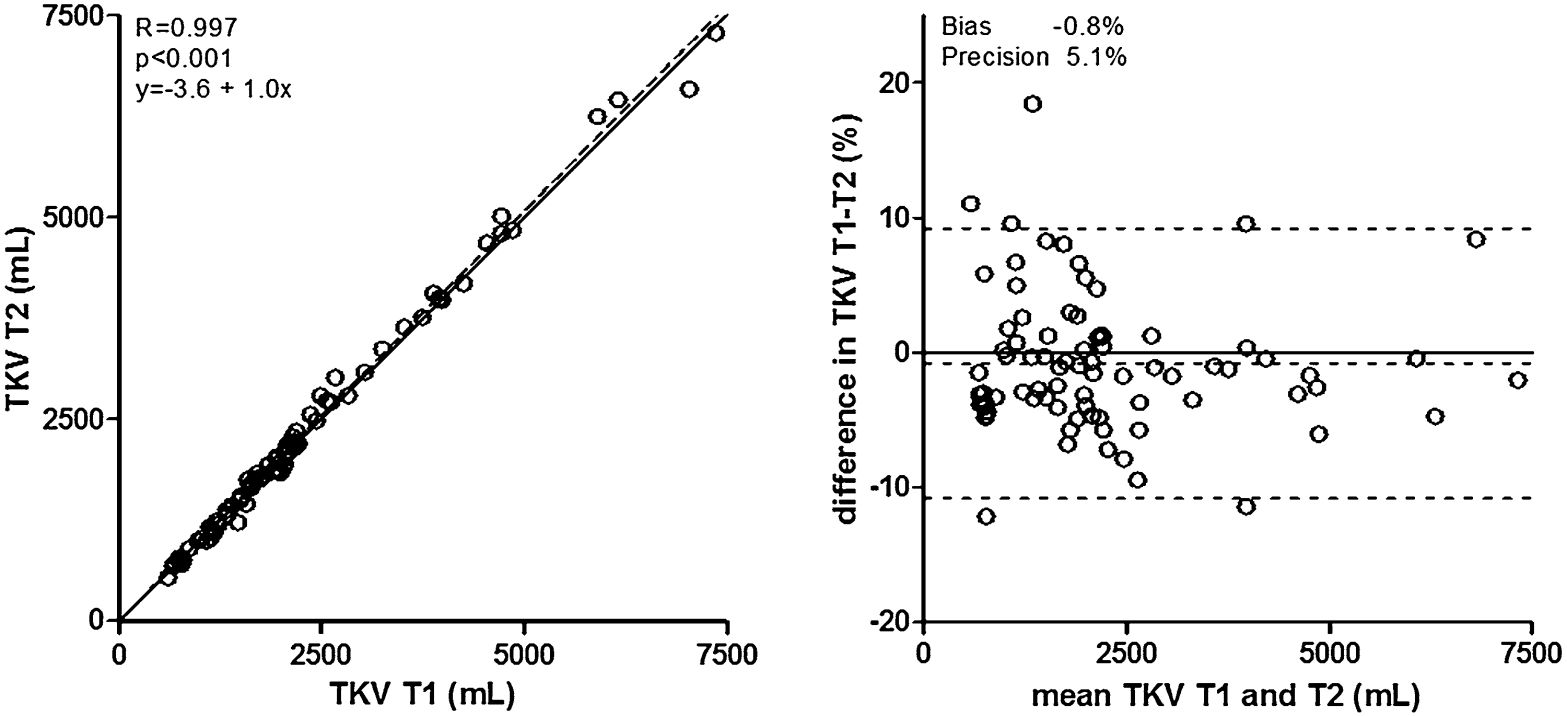
Cross-sectional associations of TKV measured using T1 or T2 weighted MR images. *Left panel* shows scatter plot, with *solid line* representing the line of identity, and *dotted line* the actual regression line. The *right panel* shows a Bland–Altman plot, with *solid line* representing no difference, and *dotted line* the actual mean difference (bias) with 95% confidence interval

The volumes measured on T1 weighted images were clinically similar, but statistically significantly different from the volumes measured on T2 weighted images; 1932 [1189–2551] mL vs. 1867 [1172–2721] mL, *P* = 0.006 (Table 2). Volumes measured using T2 and T1 weighted images showed a high correlation with an ICC of 0.997, without indication for systemic bias in the lower or higher TKV range when T2 weighted images were used instead of T1 weighted images, with a bias and precision of −0.8 and 5.1% (Fig. 2). When the various MRI scanners were studied separately, in general similar results were obtained (Supplementary Table 2). When the included subjects were stratified according to polycystic liver volume subclass or according to T1 breath-hold trigger time, again essentially similar results were obtained (Supplementary Tables 3 and 7).

**Table 2:** Differences in kidney volume, when measured using T1 or T2 weighted images

	Volumes (mL)		Differences in volume (mL) [T1–T2]			Differences in volume (%) [(T1 − T2)/average T1|T2 × 100]		
	T1	T2	Bias	Precision	*P* value	Bias	Precision	*P* value
Left kidney	1015 [657–1260]	1012 [648–1333]	−7.2	81.4	0.6	−0.2	5.7	0.7
Right kidney	846 [512–1188]	874 [513–1246]	−24.0	63.4	<0.001	−1.5	7.0	0.06
Total kidney	1932 [1180–2551]	1867 [1172–2721]	−31.2	119.2	0.006	−0.8	5.1	0.2

Values are given as median [IQR]. *P* values are calculated using a paired Wilcoxon signed-rank test for absolute differences; for percentage differences a one sample *T* test was used

### Comparing T1 and T2 weighted images to detect changes in kidney volume

**Fig. 3:**
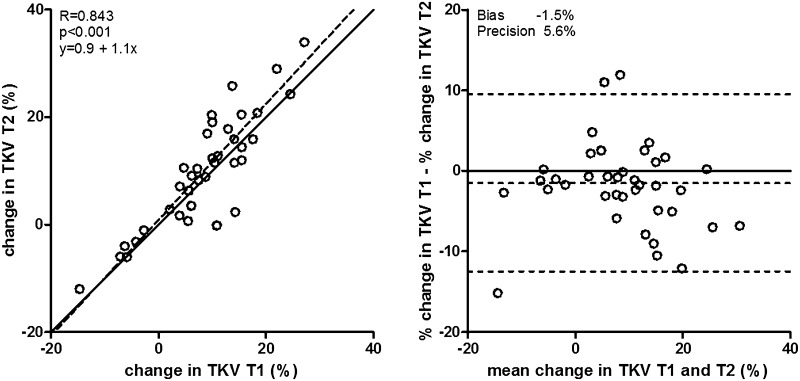
Associations of change in TKV when measured using T1 or T2 weighted MR images. *Left panel* shows scatter plot, with *solid line* representing the line of identity, and *dotted line* the actual regression line. The *right panel* shows a Bland–Altman plot, with *solid line* representing no difference, and *dotted line* the actual mean difference (bias) with 95% confidence interval

 The percentage change in TKV during follow-up was not different when comparing T1 to T2 weighted images (7.8 ± 9.9% vs. 9.3 ± 10.6%, respectively, *P* = 0.1, Table 3), and showed a high correlation (ICC 0.843, *P* < 0.001), without systematic under- or overestimation, with a bias and precision of −1.5 and 5.6% (Fig. 3). The results for the various individual MRI scanners were essentially similar to these overall results (Supplementary Table 4).

**Table 3:** Changes in kidney volumes when measured using T1 or T2 weighted images

	T1	T2	*P* value
Left kidney			
Baseline volume (mL)	971 [641–1172]	976 [591–1218]	0.9
Follow-up volume (mL)	1049 [657–1351]	1062 [654–1416]	0.3
Change (mL)	74.3 ± 131.3	92.5 ± 132.1	0.08
Change (%)	7.1 ± 11.2	8.0 ± 13.3	0.4
Right kidney			
Baseline volume (mL)	789 [526–1051]	784 [503–1136]	0.06
Follow-up volume (mL)	881 [503–1206]	896 [542–1284]	<0.001
Change (mL)	91.7 ± 131.6	111.4 ± 140.5	0.04
Change (%)	9.0 ± 11.6	11.1 ± 9.9	0.1
Total kidney			
Baseline volume (mL)	1800 [1180–2411]	1810 [1127–2621]	0.3
Follow-up volume (mL)	2055 [1166–2646]	2017 [1172–2943]	0.006
Change (mL)	166.1 ± 211.8	203.9 ± 236.4	0.02
Change (%)	7.8 ± 9.9	9.3 ± 10.6	0.1

Values are given as mean ± SD or median [IQR]. *P* values were calculated using a paired Wilcoxon signed-rank test in case of non-parametric data, in case of parametric data a paired *T* test was used

### Consequences of using T2 instead of T1 weighted images

We analyzed the consequences of using T2 instead of T1 weighted images for risk assessment according to the Mayo classification, that categorizes patients into five risk classes for disease progression based on htTKV at a given age [14]. Thirty-four out of the forty patients (85%) remained in their original risk class, whereas two patients (5%) were reclassified to a higher and four patients (10%) to a lower risk category. In addition, we analyzed the consequences for the number of subjects to be included in a clinical trial when change in TKV would be the endpoint, and when this change in TKV was to be measured with T2 instead of T1 weighted images. Power analyses indicated that a somewhat smaller patient group has to be randomized when measuring TKV on T2 weighted images (Table 4).

**Table 4:** Sample size calculation for clinical trials using T1 or T2 weighted images adopting a power of 80% and a two-sided alpha of 0.05

Assumed difference in rate of TKV growth (%)	T1 (*n*)	T2 (*n*)
30	146	113
25	214	167
20	301	245

Of all baseline MR images of the DIPAK-1 study participants (*n* = 308), the percentage of T1 and of T2 weighted images that was deemed suitable for TKV measurement was 71.1% and 86.7%, respectively (*P* < 0.001). The remaining MR images were not suitable for volume measurement, because the quality of the images was insufficient. Sensitivity analysis showed that the results were not different across the various MRI scanners (Supplementary Table 5). The time needed to assess TKV was on average 41.6 ± 13.0 min for a T1 weighted scan and 44.8 ± 16.4 min for a T2 weighted scan (*P* = 0.09).

## Discussion

TKV is an increasingly important biomarker for the assessment of disease severity and disease progression in patients with ADPKD and has recently been accepted by the FDA and the European Medicines Agency as a prognostic biomarker to select patients with ADPKD for clinical trials. The T2 weighted imaging technique has been suggested to be more reliable for kidney volume measurement in ADPKD compared to T1 weighted images without gadolinium [7]. However, to our knowledge, no studies have been performed to investigate possible differences in TKV measurement between these two MRI techniques.

Signal differences between kidney and surrounding tissues have previously been shown to be important in TKV measurement in ADPKD, as Bae et al. found that T1 weighted images without gadolinium resulted in significantly smaller kidney volumes [7]. In the present study TKV could be assessed as reliable and reproducible using T2 weighted images instead of T1 weighted images, although slightly lower intra- and interreader variability was observed for T2 weighted images. Volume and growth rate assessed on T1 and T2 weighted images showed very high correlations. The assessment time for TKV was equal for both techniques, whereas the percentage of approved scans, based on the quality of images, was higher for T2 compared to T1 weighted images.

In T1 weighted images tissues with high fat content appear hyperintense (e.g., perinephric fat) and compartments filled with water (e.g., cysts) appear hypointense, whereas the opposite is true for T2 weighted images. As a consequence T2 weighted images have a higher soft-tissue contrast and hyperintense renal cysts that help to delineate the kidney boundaries against background tissue more easily [7]. Of note, the soft-tissue contrast is dependent on whether or not fat suppression is used. In our cohort we included MR images with and without fat suppression, which was dependent on the type of scanner used. This could theoretically lead to volume differences between T1 and T2 weighted images. However, a sensitivity analysis showed similar results for the comparison of T1 vs. T2 across the various scanners that were used, which indicates that there is no major effect of fat suppression on differences in volumes between T1 and T2 weighted images. The breath-hold trigger time was less than 26 s for all T1 weighted MR images and also caused no differences in volumes measured on T1 and T2. Another factor that influences the quality of MR images is related to the content of cysts. Hemorrhagic cysts appear hyperintense compared to the surrounding cysts on T1 weighted images, whereas they are hypointense on T2 weighted images and possibly more difficult to recognize on this sequence. Hemorrhagic cysts that occur at the border of the kidney can be missed, which theoretically could affect the measured TKV on T2 weighted images. However, we did not encounter this when reviewing differences in TKV values between T1 and T2 weighted images. Lastly, although the aforementioned factors could cause differences in TKV when measured using T1 or T2 weighted images, we hypothesize that kidney volumes are probably that large in patients with ADPKD, that the aforementioned factors have no relevant effect.

We found however, that T1 weighted images resulted in statistically significant smaller kidney volumes. Nonetheless, volumes were clinically similar with a mean difference between volumes of less than one percent, which is less than the interreader CV and therefore of low clinical relevance. This assumption was confirmed when assessing the consequences for risk classification using the Mayo htTKV classification; only 15% of patients was reclassified using the kidney volumes observed on T1 vs. T2 measurements. In addition, no systemic over- or under-classification was observed. When looking in detail at the differences in TKV assessed on T1 vs. T2 weighted images, we found that the smaller T1 TKV was driven by the right kidney volume. This led us to hypothesize that the liver might have caused difficulties in distinguishing kidney from liver tissue. To corroborate our hypothesis, we therefore performed a sensitivity analysis according to the PLD classification [13]. However, we found no significant difference between the different liver volumes, but this analysis is hampered by the fact that we had only four patients in the most severe polycystic liver category.

The performance of T1 and T2 weighted images in assessing percentage kidney growth was similar. However, when calculating the number of patients needed for clinical trials that test novel renoprotective agents and change in TKV as endpoint, fewer patients were needed when using T2 instead of T1 weighted images. Moreover, when analyzing the percentage of T1 and T2 weighted MR images that were deemed suitable for volume measurement, we found that T1 weighted images were more often rejected because the quality of the images was insufficient to reliably delineate the kidney borders for volume measurement. In clinical practice this would result in making an extra MRI, with an increase in costs and patient burden as a result. This could form a rationale to only scan T2 weighted images for the assessment of kidney volume, which would save scanning time. Lastly, although TKV growth rates were similar when using T1 or T2 weighted images, one should consider using the same sequence for follow-up within one patient, to reduce measurement variations introduced using two different sequences in one patient.

Our study has the limitation that it was performed in a relatively small number of patients. However, this number of patients has previously shown to be sufficient to detect differences in total kidney volume measurement techniques in ADPKD [7]. Second, the MR images were made in patients participating in a randomized controlled trial with specific inclusion criteria for age and renal function to enrich the patient population for a high likelihood of rapid disease progression. This may limit extrapolation of our findings to the general ADPKD population.

Strengths of our study are that we had follow-up data available, enabling us to compare change in TKV, which is one of the most important parameters for disease progression in patients with ADPKD. In addition, we had three readers measuring TKV to assess intra- and interreader variability, and multiple parameters were available to assess feasibility of the two techniques, such as duration of assessment and approval rates of T1 and T2 weighted images.

In conclusion, we found that kidney volumes and kidney volume growth rates assessed on T2 and T1 weighted images were comparable. These findings show that T2 weighted images can be used, but are not superior to T1 weighted images for kidney volume measurement in patients with ADPKD. Differences between T2 and T1 were small, and likely not clinically relevant although on secondary outcome parameters T2 had minor advantages over T1 weighted images.

## Electronic supplementary material

Below is the link to the electronic supplementary material.

Supplementary material 1 (PDF 22 kb)

Supplementary material 2 (PDF 90 kb)

Supplementary material 3 (PDF 72 kb)

Supplementary material 4 (PDF 26 kb)

Supplementary material 5 (PDF 12 kb)

Supplementary material 6 (PDF 93 kb)

Supplementary material 7 (PDF 161 kb)
