# Supplementary material for: T1 vs. T2 weighted magnetic resonance imaging to assess total kidney volume in patients with autosomal dominant polycystic kidney disease
Source: Abdom Radiol (NY). 2017 Sep 4;43(5):1215–22. doi: 10.1007/s00261-017-1285-2 (PMC5904223; doi:10.1007/s00261-017-1285-2)
Supplement: Supplementary file 1 — Supplementary material 1 (PDF 22 kb) [file 261_2017_1285_MOESM1_ESM.pdf]

T1 versus T2 weighted Magnetic Resonance Imaging to Assess Total Kidney Volume in Patients with Autosomal Dominant Polycystic Kidney Disease

Journal: Abdominal Radiology

Maatje D.A. van Gastel *, BSc[1]; A. Lianne Messchendorp *, MD[1]; Peter Kappert, MSc[2]; Merel A. Kaatee, BSc[1,3]; Marissa de Jong, BSc[1]; Remco J. Renken, MSc, PhD[4]; Gert J. ter Horst, MSc, PhD[4]; Shekar V.K. Mahesh, MD[2] and Ron T. Gansevoort, MD, PhD[1].
On behalf of the DIPAK consortium

Departments of [1] Nephrology, [2]Radiology, [3]Center for Medical Imaging and [4]Neuro Imaging Center, University of Groningen, University Medical Center Groningen, Groningen, the Netherlands.

* both authors contributed equally to this work

Correspondence: Ron T. Gansevoort
Email: r.t.gansevoort@umcg.nl

Supplementary Table 1. Intra- and interreader coefficients of variability in kidney volume measurements (three readers), for 1.5 versus 3 Tesla, as well as different manufacturers.

	Intrareader CV (%)		Interreader CV (%)	
	**T1**	**T2**	**T1**	**T2**
**Left kidney**	P=0.5	P=0.1	P=0.2	P=0.9
Magneto Avanto, Siemens[1]	1.40 [0.53 - 1.64]	0.62 [0.38 - 0.83]	1.62 [1.41 - 4.42]	0.98 [0.87 - 1.25]
Ingenia, Philips[1]	1.10 [0.56 - 2.62]	0.80 [0.43 - 1.52]	1.20 [0.72 - 3.33]	0.95 [0.34 - 2.60]
GE Medical Systems[1]	0.97 [0.88 - 1.77]	0.86 [0.29 - 1.21]	3.65 [3.09 - 4.95]	1.61 [0.43 - 2.22]
3 Tesla scanner[2]	0.47 [0.15 - 1.77]	0.21 [0.08 - 0.64]	1.10 [0.92 - 1.63]	1.02 [0.98 - 1.02]
**Right kidney**	P=0.6	P=0.9	P=0.06	P=0.5
Magneto Avanto, Siemens[1]	1.78 [0.93 - 2.55]	0.81 [0.33 - 0.94]	0.59 [0.54 - 3.26]	0.76 [0.34 - 1.92]
Ingenia, Philips[1]	1.57 [1.17 - 2.14]	0.65 [0.11 - 1.68]	1.95 [0.52 - 2.00]	1.85 [0.90 - 2.41]
GE Medical Systems[1]	1.35 [0.56 - 2.05]	0.61 [0.34 - 1.40]	4.37 [4.13 - 17.27]	1.00 [0.74 - 1.31]
3 Tesla scanner[2]	0.86 [0.67 - 1.96]	0.56 [0.31 - 0.83]	3.47 [1.74 - 3.99]	1.42 [1.19 - 1.90]
**Total kidney**	P=0.2	P=0.3	P=0.09	P=0.2
Magneto Avanto, Siemens[1]	1.67 [0.96 - 2.00]	0.98 [0.42 - 1.37]	1.05 [0.85 - 3.87]	1.76 [1.04 - 2.42]
Ingenia, Philips[1]	1.04 [0.20 - 1.61]	1.57 [0.52 - 2.82]	1.53 [1.05 - 1.85]	2.67 [1.97 - 5.09]
GE Medical Systems[1]	0.83 [0.25 - 2.03]	0.79 [0.54 - 2.24]	4.64 [3.88 - 8.12]	1.54 [1.48 - 2.19]
3 Tesla scanner[2]	0.41 [0.27 - 1.41]	0.74 [0.10 - 1.24]	1.37 [1.14 - 2.09]	2.29 [2.17 - 3.37]

Values are given as median [IQR] for intrareader CV and median [minimum-maximum] for interreader CV. P values show differences between the four scanner types, using a Kruskal Wallis Test. 1. 1.5 Tesla scanner; 2. Magnetom TRIO, Siemens. *Abbreviations:* CV, coefficient of variability.
