# Supplementary material for: T1 vs. T2 weighted magnetic resonance imaging to assess total kidney volume in patients with autosomal dominant polycystic kidney disease
Source: Abdom Radiol (NY). 2017 Sep 4;43(5):1215–22. doi: 10.1007/s00261-017-1285-2 (PMC5904223; doi:10.1007/s00261-017-1285-2)
Supplement: Supplementary file 2 — Supplementary material 2 (PDF 90 kb) [file 261_2017_1285_MOESM2_ESM.pdf]

T1 versus T2 weighted Magnetic Resonance Imaging to Assess Total Kidney Volume in Patients with Autosomal Dominant Polycystic Kidney Disease

Journal: Abdominal Radiology

Maatje D.A. van Gastel *, BSc[1]; A. Lianne Messchendorp *, MD[1]; Peter Kappert, MSc[2]; Merel A. Kaatee, BSc[1,3]; Marissa de Jong, BSc[1]; Remco J. Renken, MSc, PhD[4]; Gert J. ter Horst, MSc, PhD[4]; Shekar V.K. Mahesh, MD[2] and Ron T. Gansevoort, MD, PhD[1].
On behalf of the DIPAK consortium

Departments of [1] Nephrology, [2]Radiology, [3]Center for Medical Imaging and [4]Neuro Imaging Center, University of Groningen, University Medical Center Groningen, Groningen, the Netherlands.

* both authors contributed equally to this work

Correspondence: Ron T. Gansevoort
Email: r.t.gansevoort@umcg.nl

Supplementary Table 2. Differences in kidney volume when measured using T1 or T2 weighted images for 1.5 versus 3 Tesla, as well as different manufacturers.

	Volumes (mL)		Differences in volume (mL) [T1 – T2]			Differences in volume (%) [(T1 – T2) / average T1|T2* 100]		
	**T1**	**T2**	**Bias**	**Precision**	***P value****	**Bias**	**Precision**	***P value*** #
**Left kidney**								
Magneto Avanto, Siemens[1]	1034 [484 - 2257]	1026 [460 - 2229]	10.1	115.0	0.2	1.0	6.7	0.5
Ingenia, Philips[1]	1075 [721 - 1260]	1086 [758 - 1333]	-20.5	37.1	0.03	-1.9	3.8	0.04
GE Medical Systems[1]	983 [655 - 1174]	977 [654 - 1195]	-0.2	57.1	0.6	1.2	5.2	0.3
3 Tesla scanner[2]	905 [715 - 1278]	939 [722 - 1365]	-30.3	80.9	0.1	-2.2	6.1	0.2
**Right kidney**								
Magneto Avanto, Siemens[1]	813 [353 - 1802]	853 [374 - 1851]	-36.9	72.8	0.008	-3.0	5.3	0.008
Ingenia, Philips[1]	828 [680 - 1319]	873 [681 - 1384]	-28.7	37.3	0.004	-1.8	6.0	0.2
GE Medical Systems[1]	892 [560 - 1049]	898 [513 - 1136]	-13.7	83.2	0.2	0.4	10.7	0.9
3 Tesla scanner[2]	857 [712 - 1072]	837 [729 - 1081]	-8.0	35.8	0.3	-0.9	4.0	0.4
**Total kidney**								
Magneto Avanto, Siemens[1]	1800 [785 - 4031]	1810 [785 - 4090]	-26.8	154.0	0.3	-0.7	4.7	0.4
Ingenia, Philips[1]	2008 [1418 - 2774]	2054 [1448 - 2773]	-49.2	64.1	0.005	-1.8	4.3	0.07
GE Medical Systems[1]	1883 [1186 - 2203]	1902 [1152 - 2317]	-14.0	122.8	0.7	0.8	6.4	0.8
3 Tesla scanner[2]	1798 [1461 - 2262]	1792 [1469 - 2363]	-38.2	107.8	0.08	-1.7	4.9	0.2

Values are given as median [IQR]. P values shown are: * absolute differences (mL) between T1 and T2 weighted volumes using a paired Wilcoxon signed rank test; # percentage differences (%) between T1 and T2 weighted volumes using a one sample T-test. ANOVA showed no significant difference between the scanners for absolute and percentage difference. 1. 1.5 Tesla scanner; 2. Intera, Philips and Magnetom TRIO, Siemens.
