# Supplementary material for: T1 vs. T2 weighted magnetic resonance imaging to assess total kidney volume in patients with autosomal dominant polycystic kidney disease
Source: Abdom Radiol (NY). 2017 Sep 4;43(5):1215–22. doi: 10.1007/s00261-017-1285-2 (PMC5904223; doi:10.1007/s00261-017-1285-2)
Supplement: Supplementary file 3 — Supplementary material 3 (PDF 72 kb) [file 261_2017_1285_MOESM3_ESM.pdf]

# T1 versus T2 weighted Magnetic Resonance Imaging to Assess Total Kidney Volume in Patients with Autosomal Dominant Polycystic Kidney Disease

*Journal: Abdominal Radiology*

Maatje D.A. van Gastel *, BSc[1]; A. Lianne Messchendorp *, MD[1]; Peter Kappert, MSc[2]; Merel A. Kaatee, BSc[1,3]; Marissa de Jong, BSc[1]; Remco J. Renken, MSc, PhD[4]; Gert J. ter Horst, MSc, PhD[4]; Shekar V.K. Mahesh, MD[2] and Ron T. Gansevoort, MD, PhD[1].
On behalf of the DIPAK consortium

Departments of [1] Nephrology, [2]Radiology, [3]Center for Medical Imaging and [4]Neuro Imaging Center, University of Groningen, University Medical Center Groningen, Groningen, the Netherlands.

* both authors contributed equally to this work

**Correspondence:** Ron T. Gansevoort
**Email:** r.t.gansevoort@umcg.nl

**Supplementary Table 3.** Differences in TKV between T1 and T2 stratified according to liver volume (PLD classification) [1].

| | Mild (n=60) (htTLV<1600 mL/m) | | Moderate (n=16) (htTLV≥1600 and <3200 mL/m) | | Severe (n=4) (htTLV≥3200 mL/m) | |
|---|---|---|---|---|---|---|
| | **T1** | **T2** | **T1** | **T2** | **T1** | **T2** |
| **Left kidney volume** | 1035 [636 - 1451] | 1048 [629 - 1659] | 986 [685 - 1236] | 984 [678 - 1253] | 688 [668 - 707] | 650 [607 - 700] |
| **Right kidney volume** | 919 [622 - 1320] | 921 [610 - 1387]* | 730 [577 - 1006] | 748 [574 - 1064]* | 433 [316 - 689] | 441 [360 - 499] |
| **Total kidney volume** | 1989 [1238 - 2789] | 1951 [1257 - 2961]* | 1716 [1271 - 2160] | 1732 [1257 - 2289]* | 1112 [1001 - 1388] | 1065 [993 - 1199] |

Values are given as median [IQR]. *Abbreviations:* htTLV, height adjusted total liver volume. * $p<0.05$
