# Supplementary material for: T1 vs. T2 weighted magnetic resonance imaging to assess total kidney volume in patients with autosomal dominant polycystic kidney disease
Source: Abdom Radiol (NY). 2017 Sep 4;43(5):1215–22. doi: 10.1007/s00261-017-1285-2 (PMC5904223; doi:10.1007/s00261-017-1285-2)
Supplement: Supplementary file 4 — Supplementary material 4 (PDF 26 kb) [file 261_2017_1285_MOESM4_ESM.pdf]

T1 versus T2 weighted Magnetic Resonance Imaging to Assess Total Kidney Volume in Patients with Autosomal Dominant Polycystic Kidney Disease

Journal: Abdominal Radiology

Maatje D.A. van Gastel *, BSc[1]; A. Lianne Messchendorp *, MD[1]; Peter Kappert, MSc[2]; Merel A. Kaatee, BSc[1,3]; Marissa de Jong, BSc[1]; Remco J. Renken, MSc, PhD[4]; Gert J. ter Horst, MSc, PhD[4]; Shekar V.K. Mahesh, MD[2] and Ron T. Gansevoort, MD, PhD[1].
On behalf of the DIPAK consortium

Departments of [1] Nephrology, [2]Radiology, [3]Center for Medical Imaging and [4]Neuro Imaging Center, University of Groningen, University Medical Center Groningen, Groningen, the Netherlands.

* both authors contributed equally to this work

Correspondence: Ron T. Gansevoort
Email: r.t.gansevoort@umcg.nl

Supplementary Table 4. Kidney growth (change in kidney volumes) for 1.5 versus 3 Tesla, as well as different manufacturers.

	T1	T2	*P value*
**Left kidney**			
Change (mL)			
Magneto Avanto, Siemens[1]	28.4 ± 131.4	67.6 ± 110.6	0.2
Ingenia, Philips[1]	119.8 ± 188.3	128.9 ± 190.5	0.4
GE Medical Systems[1]	72.2 ± 99.9	81.7 ± 108.6	0.6
3 Tesla scanner	83.3 ± 16.1	144.5 ± 70.2	0.1
Change (%)			
Magneto Avanto, Siemens[1]	5.1 ± 7.1	5.3 ± 9.8	0.9
Ingenia, Philips[1]	9.4 ± 16.0	10.1 ± 16.2	0.5
GE Medical Systems[1]	6.8 ± 12.1	8.9 ± 14.9	0.4
3 Tesla scanner[2]	10.1 ± 2.7	17.4 ± 6.9	0.1
**Right kidney**			
Change (mL)			
Magneto Avanto, Siemens[1]	137.6 ± 198.0	141.0 ± 240.7	0.9
Ingenia, Philips[1]	95.6 ± 102.8	114.1 ± 120.0	0.1
GE Medical Systems[1]	55.0 ± 137.7	88.1 ± 88.1	0.3
3 Tesla scanner[2]	91.2 ± 19.7	130.7 ± 46.0	0.06
Change (%)			
Magneto Avanto, Siemens[1]	13.3 ± 12.9	12.9 ± 11.6	0.9
Ingenia, Philips[1]	9.1 ± 7.8	11.3 ± 10.0	0.1
GE Medical Systems[1]	5.0 ± 17.5	9.4 ± 12.0	0.3
3 Tesla scanner[2]	11.0 ± 2.2	16.1 ± 5.2	0.07
**Total kidney**			
Change (mL)			
Magneto Avanto, Siemens[1]	166.0 ± 233.6	208.5 ± 296.1	0.3
Ingenia, Philips[1]	215.5 ± 268.0	243.0 ± 291.1	0.1
GE Medical Systems[1]	127.2 ± 223.9	169.7 ± 195.7	0.3
3 Tesla scanner[2]	174.5 ± 30.9	275.1 ± 112.1	0.09
Change (%)			
Magneto Avanto, Siemens[1]	8.3 ± 7.4	8.3 ± 8.2	0.9
Ingenia, Philips[1]	9.3 ± 11.3	10.8 ±12.6	0.1
GE Medical Systems[1]	5.8 ± 14.2	9.0 ± 13.4	0.2
3 Tesla scanner[2]	10.4 ± 1.8	16.5 ± 5.2	0.08

Values are given as mean ± standard deviation or median [IQR]. P values were calculated using a paired Wilcoxon signed rank test for differences between T1 and T2 weighted volumes, for differences in change in volumes between T1 and T2 a paired T-test was used. ANOVA showed no significant difference between any of the four scanners. 1. 1.5 Tesla scanner; 2. Intera, Philips and Magnetom TRIO, Siemens.
