# Supplementary material for: T1 vs. T2 weighted magnetic resonance imaging to assess total kidney volume in patients with autosomal dominant polycystic kidney disease
Source: Abdom Radiol (NY). 2017 Sep 4;43(5):1215–22. doi: 10.1007/s00261-017-1285-2 (PMC5904223; doi:10.1007/s00261-017-1285-2)
Supplement: Supplementary file 5 — Supplementary material 5 (PDF 12 kb) [file 261_2017_1285_MOESM5_ESM.pdf]

T1 versus T2 weighted Magnetic Resonance Imaging to Assess Total Kidney Volume in Patients with Autosomal Dominant Polycystic Kidney Disease

Journal: Abdominal Radiology

Maatje D.A. van Gastel *, BSc[1]; A. Lianne Messchendorp *, MD[1]; Peter Kappert, MSc[2]; Merel A. Kaatee, BSc[1,3]; Marissa de Jong, BSc[1]; Remco J. Renken, MSc, PhD[4]; Gert J. ter Horst, MSc, PhD[4]; Shekar V.K. Mahesh, MD[2] and Ron T. Gansevoort, MD, PhD[1].
On behalf of the DIPAK consortium

Departments of [1] Nephrology, [2]Radiology, [3]Center for Medical Imaging and [4]Neuro Imaging Center, University of Groningen, University Medical Center Groningen, Groningen, the Netherlands.

* both authors contributed equally to this work

Correspondence: Ron T. Gansevoort
Email: r.t.gansevoort@umcg.nl

Supplementary Table 5. Percentage of approved scans when using T1 or T2 weighted images per MRI scanner.

	**T1 [n (%)]**	**T2 [n (%)]**	***P value***
Magneto Avanto, Siemens[1]	37 (64.9)	51 (89.5)	0.002
Ingenia, Philips[1]	57 (74.0)	70 (90.9)	0.006
GE Medical Systems[1]	49 (75.4)	55 (84.6)	0.19
3 Tesla scanner[2]	76 (69.7)	91 (83.5)	0.02
P value	0.6	0.4	

Values represent the number of MR images suitable for volume measurement. P values were calculated using a Chi-square test. 1. 1.5 Tesla scanner; 2. Intera, Philips and Magnetom TRIO, Siemens.
