# Supplementary material for: T1 vs. T2 weighted magnetic resonance imaging to assess total kidney volume in patients with autosomal dominant polycystic kidney disease
Source: Abdom Radiol (NY). 2017 Sep 4;43(5):1215–22. doi: 10.1007/s00261-017-1285-2 (PMC5904223; doi:10.1007/s00261-017-1285-2)
Supplement: Supplementary file 6 — Supplementary material 6 (PDF 93 kb) [file 261_2017_1285_MOESM6_ESM.pdf]

T1 versus T2 weighted Magnetic Resonance Imaging to Assess Total Kidney Volume in Patients with Autosomal Dominant Polycystic Kidney Disease

Journal: Abdominal Radiology

Maatje D.A. van Gastel *, BSc[1]; A. Lianne Messchendorp *, MD[1]; Peter Kappert, MSc[2]; Merel A. Kaatee, BSc[1,3]; Marissa de Jong, BSc[1]; Remco J. Renken, MSc, PhD[4]; Gert J. ter Horst, MSc, PhD[4]; Shekar V.K. Mahesh, MD[2] and Ron T. Gansevoort, MD, PhD[1].
On behalf of the DIPAK consortium

Departments of [1] Nephrology, [2]Radiology, [3]Center for Medical Imaging and [4]Neuro Imaging Center, University of Groningen, University Medical Center Groningen, Groningen, the Netherlands.

* both authors contributed equally to this work

Correspondence: Ron T. Gansevoort
Email: r.t.gansevoort@umcg.nl

Supplementary Table 6. Intra- and interreader coefficients of variability in kidney volume measurements according to quartiles of T1 breath-hold trigger time.

	Intrareader CV (%)		Interreader CV (%)	
	**T1**	**T2**	**T1**	**T2**
**Left kidney**	P=0.6	P=0.1	P=0.5	P=0.5
16.5 – 17.2 sec.	1.59 [1.10 - 2.26]	0.54 [0.40 - 0.82]	3.02 [1.62 - 4.42]	0.93 [0.87 – 0.98]
17.3 – 17.6 sec.	0.69 [0.56 - 3.64]	0.73 [0.22 - 0.88]	2.37 [1.41 - 3.33]	0.79 [0.34 – 1.25]
17.7 – 18.5 sec.	1.10 [0.62 - 1.45]	1.14 [0.50 - 1.93]	1.19 [0.72 - 3.65]	2.21 [0.95 - 2.60]
18.6 – 19.5 sec.	0.94 [0.83 - 1.95]	0.58 [0.41 - 1.16]	4.02 [3.09 - 4.95]	1.02 [0.43 - 1.61]
**Right kidney**	P=0.3	P=0.9	P=0.1	P=0.8
16.5 – 17.2 sec.	1.95 [0.97 - 3.06]	0.78 [0.25 - 1.01]	1.90 [0.54 - 3.26]	1.34 [0.76 - 1.92]
17.3 – 17.6 sec.	1.73 [1.06 - 2.21]	0.65 [0.15 - 1.25]	0.56 [0.52 - 0.59]	1.37 [0.34 - 2.41]
17.7 – 18.5 sec.	1.20 [0.44 – 1.64]	1.07 [0.14 - 1.40]	2.00 [1.95 - 4.13]	1.31 [0.90 - 1.85]
18.6 – 19.5 sec.	1.84 [0.61 – 5.23]	0.57 [0.41 - 1.16]	10.82 [4.37-17.27]	0.87 [0.74 - 1.00]
**Total kidney**	P=0.1	P=0.3	P=0.2	P=0.9
16.5 – 17.2 sec.	1.84 [1.41 - 2.17]	0.75 [0.10 - 1.53]	2.46 [1.05 - 3.87]	2.09 [1.76 - 2.42]
17.3 – 17.6 sec.	1.16 [0.55 - 1.86]	1.02 [0.86 - 1.63]	1.35 [0.85 - 1.85]	1.85 [1.04 - 2.67]
17.7 – 18.5 sec.	0.83 [0.19 – 1.54]	1.57 [0.40 - 2.87]	1.53 [1.05 - 3.88]	1.97 [1.54 - 5.09]
18.6 – 19.5 sec.	1.30 [0.25 - 2.94]	0.81 [0.56 - 2.30]	6.38 [4.64 - 8.12]	1.83 [1.48 - 2.19]

Values are given as median [IQR] for intrareader CV and median [minimum-maximum] for interreader CV. P values show differences between the quartiles of T1 breath-hold trigger time, using a Kruskal Wallis Test. *Abbreviations:* CV, coefficient of variability; sec., seconds.
