# Supplementary material for: T1 vs. T2 weighted magnetic resonance imaging to assess total kidney volume in patients with autosomal dominant polycystic kidney disease
Source: Abdom Radiol (NY). 2017 Sep 4;43(5):1215–22. doi: 10.1007/s00261-017-1285-2 (PMC5904223; doi:10.1007/s00261-017-1285-2)
Supplement: Supplementary file 7 — Supplementary material 7 (PDF 161 kb) [file 261_2017_1285_MOESM7_ESM.pdf]

T1 versus T2 weighted Magnetic Resonance Imaging to Assess Total Kidney Volume in Patients with Autosomal Dominant Polycystic Kidney Disease

Journal: Abdominal Radiology

Maatje D.A. van Gastel *, BSc[1]; A. Lianne Messchendorp *, MD[1]; Peter Kappert, MSc[2]; Merel A. Kaatee, BSc[1,3]; Marissa de Jong, BSc[1]; Remco J. Renken, MSc, PhD[4]; Gert J. ter Horst, MSc, PhD[4]; Shekar V.K. Mahesh, MD[2] and Ron T. Gansevoort, MD, PhD[1].
On behalf of the DIPAK consortium

Departments of [1] Nephrology, [2]Radiology, [3]Center for Medical Imaging and [4]Neuro Imaging Center, University of Groningen, University Medical Center Groningen, Groningen, the Netherlands.

* both authors contributed equally to this work

Correspondence: Ron T. Gansevoort
Email: r.t.gansevoort@umcg.nl

Supplementary Table 7. Differences in kidney volume when measured using T1 or T2 weighted images for quartiles of breath hold trigger time of T1 weighted images.

	Volumes (mL)		Differences in volume (mL) [T1 – T2]			Differences in volume (%) [(T1 – T2) / average T1|T2* 100]		
	**T1**	**T2**	**Bias**	**Precision**	***P value****	**Bias**	**Precision**	***P value*** #
**Left kidney**								
5.2 – 15.0 sec.	940 [584 - 1127]	951 [534 - 1110]	16.7	46.3	0.2	2.1	5.7	0.2
15.1 – 17.5 sec.	711 [394 - 1265]	716 [403 - 1357]	38.7	105.3	0.2	1.9	5.4	0.2
17.6 – 18.5 sec.	1149 [1042 - 1775]	1195 [994 - 1821]	-24.9	55.4	0.1	-1.9	4.4	0.1
18.6 – 26.0 sec.	971 [655 - 1220]	979 [654 - 1206]	-3.0	33.6	0.9	0.3	3.4	0.8
**Right kidney**								
5.2 – 15.0 sec.	845 [526 - 1038]	873 [521 - 1035]	4.2	41.2	0.4	1.1	5.3	0.4
15.1 – 17.5 sec.	729 [358 - 1988]	505 [347 - 2032]	-18.6	109.5	0.1	-0.6	12.8	0.9
17.6 – 18.5 sec.	1030 [801 - 1553]	1107 [835 - 1581]	-42.8	47.9	0.003	-3.4	3.6	0.002
18.6 – 26.0 sec.	780 [497 - 1141]	812 [498 - 1205]	-21.5	35.2	0.02	-1.5	5.6	0.3
**Total kidney**								
5.2 – 15.0 sec.	1932 [1139 - 2138]	1851 [1042 - 2194]	20.9	82.1	0.3	1.6	5.0	0.2
15.1 – 17.5 sec.	1469 [766 - 3031]	1221 [744 - 3084]	20.2	170.5	0.7	1.1	6.7	0.5
17.6 – 18.5 sec.	2130 [1976 - 3147]	2246 [1961 - 3226]	-67.6	90.9	0.01	-2.6	3.2	0.005
18.6 – 26.0 sec.	1800 [1166 - 2368]	1810 [1152 - 2394]	-24.5	59.5	0.2	-0.6	3.9	0.6

Values are given as median [IQR]. P values shown are: * absolute differences (mL) between T1 and T2 weighted volumes using a paired Wilcoxon signed rank test; # percentage differences (%) between T1 and T2 weighted volumes using a one sample T-test. ANOVA showed no significant P for trend between quartiles of breath hold trigger time of T1 for absolute and percentage difference.
